# Serological survey and associated risk factors of Aujeszky’s disease virus in wild boar from south and central Poland

**DOI:** 10.2478/jvetres-2025-0031

**Published:** 2025-06-09

**Authors:** Anna Didkowska, Daniel Klich, Katarzyna Matusik, Ewelina Kwiecień, Wiktoria Tchórz, Mirosław Welz, Bartosz Skibniewski, Piotr Kwieciński, Michał Mierkiewicz, Krzysztof Anusz

**Affiliations:** 1 Department of Food Hygiene and Public Health Protection, Institute of Veterinary Medicine, Warsaw University of Life Sciences (SGGW), 02-787 Warsaw, Poland; 2 Department of Animal Genetics and Conservation, Institute of Animal Sciences, Warsaw University of Life Sciences (WULS-SGGW), 02-786 Warsaw, Poland; 3 Department of Preclinical Sciences, Faculty of Veterinary Medicine, Warsaw University of Life Sciences, 02-786 Warsaw; 4 Provincial Veterinary Inspectorate, 38-400 Krosno, Poland; 5 Laboratory of Molecular Biology, Vet-Lab Brudzew, 62-720 Brudzew, Poland; 6 Provincial Veterinary Inspectorate, 60-166 Poznań, Poland

**Keywords:** Aujeszky’s disease virus, Europe, risk factors, wildlife

## Abstract

**Introduction:**

Aujeszky’s disease is caused by suid herpesvirus-1, also called Aujeszky’s disease virus (ADV). The main reservoir host is the wild boar (*Sus scrofa*). The last data about ADV seroprevalence in wild boar in Poland came from over 10 years ago. There is a gap in knowledge about the current epidemiological situation. Therefore, this study aimed to characterise ADV seroprevalence and risk factors in hunted wild boar in south and central Poland.

**Material and Methods:**

Between February and June 2024, blood samples were collected from 320 wild boar (143 females and 177 males). Total antibodies to ADV were detected by a commercial indirect ELISA kit. The results were statistically analysed.

**Results:**

Antibodies against ADV were detected in serum samples from 103/320 animals (32.19%). The wild boars were more likely to be ADV seropositive with age, but the uncertainty of this prediction increased with age. Sex and location of animals did not influence ADV seroprevalence.

**Conclusion:**

The study revealed that the wild boar is still an important ADV reservoir in Poland. Even though Poland has ADV-free status, the results indicate that the situation in wild boar should be monitored. There is the risk of spillover of ADV from wild boar to domestic pig herds, potentially interfering with the control programme in Poland. The ADV seroprevalence in wild boar population shows not only a risk of the virus’ reintroduction into pig herds but also a potential threat to other domestic and wild mammals.

## Introduction

Aujeszky’s disease virus (ADV), also called suid herpesvirus-1 or pseudorabies virus, belongs to the *Herpesviridae* family, *Alphaherpesvirinae* subfamily and *Varicellovirus* genus (22, 30). The virus mainly causes neurological disorders because of its high neurotropism, doing so primarily in its swine natural hosts, but also in other mammals (33). The clinical signs in swine range from respiratory and reproductive disorders to neurological disorders and death (especially in young animals). After recovery, animals become latent carriers (33). A high range of clinical symptoms, *e.g*. tremors, convulsions, incoordination and paralysis, are noted in non-swine animals (19).

It is not only the domestic pig which is a natural host for ADV; the wild boar (*Sus scrofa*) also is. The worldwide distribution of this species, its fast reproductive rate, great adaptation potential and complex social behaviour make this species an almost ideal reservoir (25, 32). Aujeszky’s disease (AD) can sporadically occur in free-living wild boar with higher individual susceptibility or when triggered by social stress or environmental conditions (17).

Aujeszky’s disease is a compulsorily notifiable disease in EU member states: under the Animal Health Law, ADV is a category C disease (10). Most EU countries have developed official control and eradication programs (11), and Poland has done so with success, having been an AD-free country since 2023 (13). The AD-free status of a member state or region may be maintained despite proven ++AD cases in wild boar, as long as measures are in place that prevent transmission from wild boar to domestic pigs (11). However, the presence of ADV in wild boar populations represents a risk of its reintroduction into pig herds and other domestic and wild mammals (24).

The last data about ADV seroprevalence in wild boar in Poland came from 2011–2014 (23). There is a gap in knowledge about the current epidemiological situation. Therefore, our study aimed to characterise ADV seroprevalence and risk factors in hunted wild boar in southern and central Poland.

## Material and Methods

### Material

**Fig. 1.**
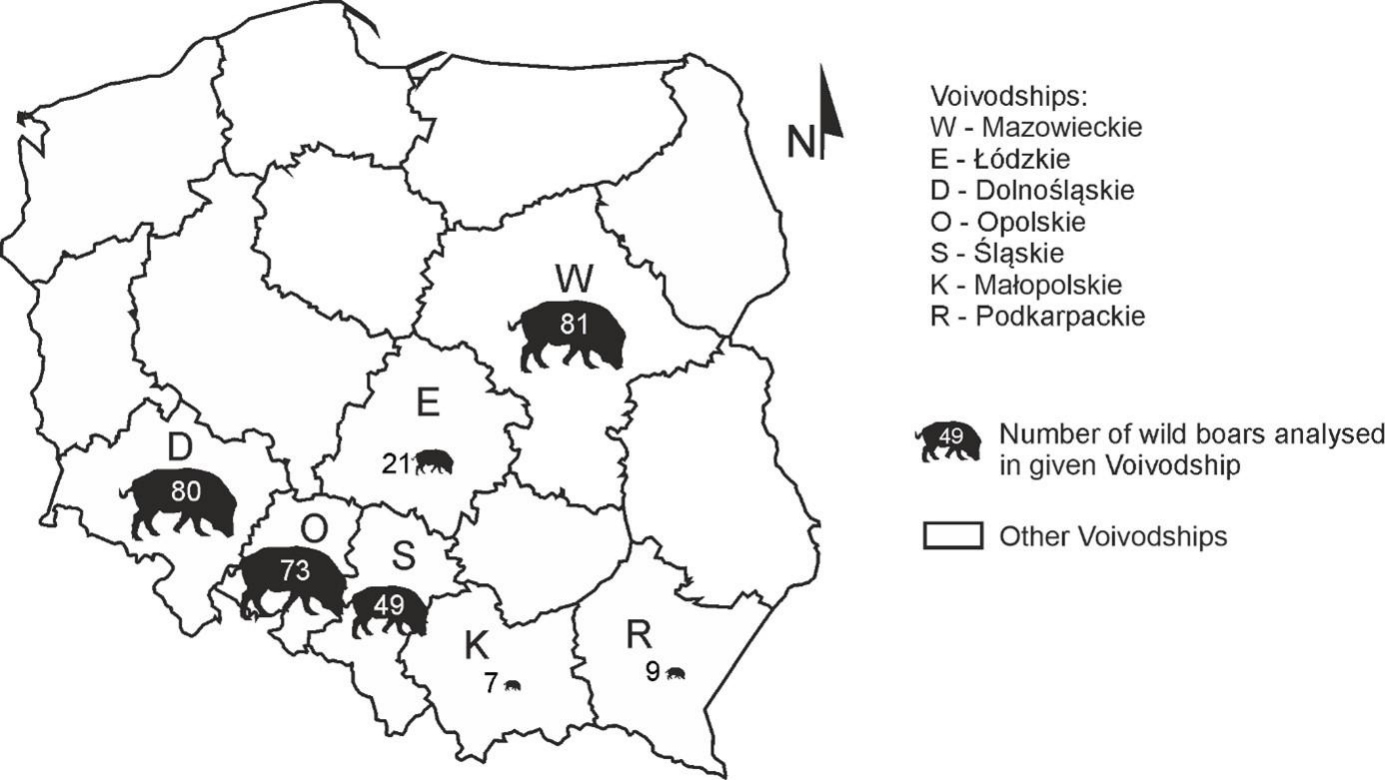
Number of wild boars analysed by voivodeship in Poland

Between February and June 2024, blood samples were collected from 320 wild boar (143 females and 177 males) from central and southern Poland. The age of the animals varied from 6 months to 4.5 years, and was on average 1.61 years. The wild boar came from seven voivodeships: Mazowieckie, Łódzkie, Dolnośląskie, Opolskie, Śląskie, Małopolskie and Podkarpackie (Fig. 1). Specimens were collected mostly from animals hunted in accordance with the hunting regulations in Poland and also from animals found dead (mainly because of being struck by a vehicle). The approval of the ethics committee was not required for this study, no live animals were used, and none were killed for the purpose of this study. Samples from hunted animals were provided by hunters with appropriate wild boar hunting licenses. A non-probabilistic sampling method (conveniencesampling) was used in this study.

Blood samples were collected from the heart postmortem. Blood was collected with a tube with a clot activator and transported to the laboratory at 4°C. After centrifugation, the serum was separated. The obtained serum samples were stored in a freezer at –20°C until further analysis.

### Methods

Before testing, serum samples were allowed to thaw at room temperature. After that, the total content of antibodies to ADV was detected by indirect ELISA (INgezim ADV TOTAL; Ingenasa, Madrid, Spain). The plates in this ELISA are coated with a soluble protein extract from the virus containing all the antigenic proteins of vaccine and field strains. The test was performed according to the manufacturer’s instructions. The results of the ELISA were read using an EPOCH spectrophotometer (BioTek Instruments, Winooski, VT, USA) at a wavelength of 450 nm and calculated following the manufacturer’s instructions.

### Statistical analysis

Calculations were made of 95% confidence limits (95% CI) using Sterne’s exact method for the prevalence of general antibodies to ADV. Then, the potential impact of sex, age and location on antibody occurrence in wild boar was analysed. For this purpose, a generalised linear model with a binary response variable was built, where the dependent variable was the test result, *i.e*. seropositive results were marked as 1 and seronegative results were marked as 0. The covariates from the model were two qualitative and one quantitative variable: the qualitative ones were the sex of the animals and the location (seven voivodeships as categories) and the quantitative one was age (expressed in years). Model selection with all model variants was performed, including the null (intercept only) model (6). We selected the highest-ranked model from various candidate models based on Akaike information criterion (AIC).

## Results

**Fig. 2.**
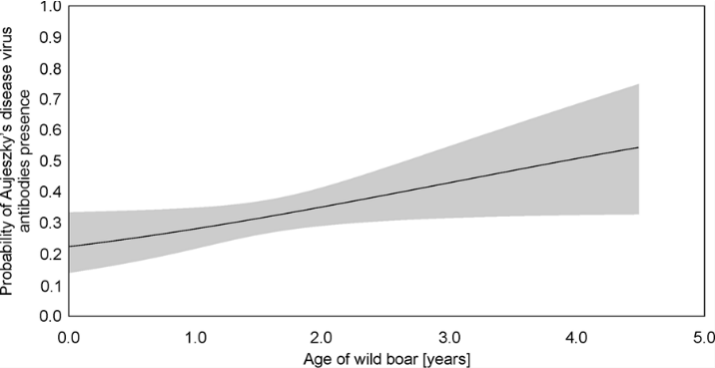
Probability of Aujeszky’s disease virus antibody presence in wild boar in given (based on the highest ranked model). For AGE: β = 0.32, standard error (SE) = 0.16, χ^2^ = 4.14, P-value = 0.042. For intercept: β = -1.27, SE = 0.29, χ^2^ = 19.29, P-value < 0.001)

Antibodies against ADV were detected in sera from 103/320 animals (32.19%; 95% CI, 27.17 to 37.48). Sex and location of animals did not influence seroprevalence and these two variables were excluded from the model (Table 1). The highest-ranked model only included age, but the explanatory power of the model was weak since ΔAIC with the null model was only 2.2 (Table 1). The only model where ΔAIC = 2 with the highest-ranked model was the variant including sex and age. However, sex was not significant in this model, and the model with it only presented a higher AIC value than the null (intercept only) model (Table 1). The wild boar were more likely to be seropositive with Aujeszky’s disease virus with age (Fig. 2), but the uncertainty of this prediction increased with age.

**Table 1.** Ranking of the models (full model selection including null model) explaining the presence of Aujeszky’s disease virus antibodies in wild boar with sex, age, and location in the generalised linear binary model

Model	ΔAIC	Ωi	Rank
Age	0.0	0.37	1
Age + sex	1.2	0.20	2
Null model	2.2	0.12	3
Age + location	2.4	0.11	4
Sex	3.3	0.07	5
Age + sex + location	3.8	0.05	6
Location	4.0	0.05	7
Sex + location	5.3	0.02	8

1 ΔAIC– AIC differences; ωi – Akaike weights; Rank – rank of the models (based on AIC values); bolded text in the row indicates the chosen model

## Discussion

The ADV seroprevalence in wild boars represents a risk factor for disease common to wildlife and livestock (17). Consequently, wildlife health surveillance has become a crucial matter, particularly concerning infectious diseases under eradication and control plans, such as AD. Our study supplied data as part of AD surveillance and revealed 32.19% ADV seroprevalence in wild boar from south and central Poland. The last report from Poland (2011–2014) showed very similar seroprevalence of 32.2% (23). Between 2011 and 2014, the Polish wild boar population was larger than it is currently. The number of wild boar has decreased after action to limit the spread of African swine fever (ASF), including culling (15, 18). The most significant decline in the population occurred between 2017 and 2018 and was from ~198,000 to ~82,000 individuals in Poland (20). Since the relative abundance and aggregation of wild boar influence the epidemiological risk, including the risk of AD (1), we could expect lower seroprevalence in our study. Nevertheless, we observed similar ADV seroprevalence in wild boars to that in the results obtained ten years ago, which is alarming. There is a risk that as the wild boar population grows, the frequency of AD will increase and its prevalence will be higher than at present.

Similarly to our results, around 30% seroprevalence was recently noted in southern Italy (14) and Croatia (21). Aujeszky’s disease virus seroprevalence in Poland is different to the seroprevalence in some other European countries and regions. It ranged from 0% in the Netherlands (10) to over 50% in Croatia and central Italy (34) and to even 100% at local level in Spain (4). However, for many countries, there are no current data.

Although seroprevalence in Poland remains stable at a level of approximately 32%, this figure shows that there is still the risk of spillover of ADV from wild boar to domestic pig herds, potentially interfering with the control programme in Poland (5). What is more, an additional risk of spillover exists from wild boar to carnivores such as hunting dogs, or endangered carnivores such as the Eurasian lynx (*Lynx lynx*) or grey wolf (*Canis lupus*) (9, 16, 24, 27). Further transmission of AD *via* predators is also a potential risk, as it has been shown that after the collapse of the wild boar population, wolf attacks on domestic animals increased significantly in Poland (20). Also, it has to be taken into consideration that concern about ADV infection in humans arises because of occasional controversial cases of encephalitis (2).

In previous studies, the highest seroprevalence was noted on the western border of Poland, where the highest density of wild boar population is (23), which could not be confirmed in our study. The continuous parallel increase of ADV seroprevalence and wild boar density was statistically correlated in previous studies (31); however, it has not been confirmed in our study, where there was no correlation with location and we did not include all western voivodeships.

Our study reveals that wild boar were more likely to be ADV seropositive with age, which is in line with previous studies (7, 14, 28, 31). In our study, there was no significant relation between sex and ADV seropositivity, which is in line with results obtained by Ferrara *et al*. (14). However, most studies indicated higher ADV exposure of females than males (3, 8). This has been explained by the more frequent spread of AD inside the maternal group. We should note that high hunting pressure can significantly impact the population structure of wild boar (26). It is likely that a sex effect was not found in our study because of hunting’s effect on the Polish population.

## Conclusion

Our data suggest that wild boar populations in south and central Poland are not free from AD. Aujeszky’s disease virus circulating in wild boar appears to be a potential source of infection for domestic pigs, as well as domestic and wild carnivores. Therefore, our data indicates the necessity for the continuance of serosurveys to assess and monitor the prevalence of ADV infection among wild boar in Poland.
